# Pan‐cancer analysis identifies the IRF family as a biomarker for survival prognosis and immunotherapy

**DOI:** 10.1111/jcmm.18084

**Published:** 2023-12-21

**Authors:** Hua‐Guo Xu, Can Chen, Lin‐Yuan Chen, Shiyang Pan

**Affiliations:** ^1^ Department of Laboratory Medicine The First Affiliated Hospital of Nanjing Medical University Nanjing China; ^2^ Branch of National Clinical Research Center for Laboratory Medicine Nanjing China

**Keywords:** immunotherapy, IRF family, pan‐cancer, targeted therapy, tumour microenvironment

## Abstract

IRF family genes have been shown to be crucial in tumorigenesis and tumour immunity. However, information about the role of IRF in the systematic assessment of pan‐cancer and in predicting the efficacy of tumour therapy is still unknown. In this work, we performed a systematic analysis of IRF family genes in 33 tumour samples, including expression profiles, genomics and clinical characteristics. We then applied Single‐Sample Gene‐Set Enrichment Analysis (ssGSEA) to calculate IRF‐scores and analysed the impact of IRF‐scores on tumour progression, immune infiltration and treatment efficacy. Our results showed that genomic alterations, including SNPs, CNVs and DNA methylation, can lead to dysregulation of IRFs expression in tumours and participate in regulating multiple tumorigenesis. IRF‐score expression differed significantly between 12 normal and tumour samples and the impact on tumour prognosis and immune infiltration depended on tumour type. IRF expression was correlated to drug sensitivity and to the expression of immune checkpoints and immune cell infiltration, suggesting that dysregulation of IRF family expression may be a critical factor affecting tumour drug response. Our study comprehensively characterizes the genomic and clinical profile of IRFs in pan‐cancer and highlights their reliability and potential value as predictive markers of oncology drug efficacy. This may provide new ideas for future personalized oncology treatment.

## 1 INTRODUCTION

Targeted therapy and immunotherapy, as emerging forms of oncology treatment, have become mainstream treatment options for many types of tumours. Targeted therapies deliver precise strikes against well‐defined cancer‐causing alterations, directly killing tumour cells^1^; while immunotherapy mobilizes the immune system's activity, restores the body's normal anti‐tumour immune response and indirectly destroys tumour cells.^2^ However, there are limitations to both approaches. For targeted therapies, due to tumour heterogeneity and evolution, most patients will develop drug resistance after a period of treatment.^3^ The slow and ineffective onset of immunotherapy means that some tumours do not respond to immunotherapy drugs in the first place.^4^ Therefore, the search for a marker that can predict the efficacy of these treatments in oncology patients is urgent.

Interferons (IFNs) are a class of cytokines capable of resisting viral infection, inhibiting the cell cycle and exercising immunomodulatory functions.^5^, ^6^, ^7^ Members of the interferon regulatory factor family (IRF) are named for their ability to bind to the IFN promoter to induce and regulate IFN expression during viral infection.^8^, ^9^ Available studies have demonstrated the role of IRFs as transcription factors in regulating interferon transcription, signal transduction, and modulating innate and adaptive immunity.^10^, ^11^, ^12^ IRF family has been found to predict multiple tumour prognosis and the efficacy of interventional therapy.^13^ For example, IRF1,^14^ IRF2,^15^ IRF3,^16^ IRF4,^17^ IRF7^18^ and IRF8^19^ intervene in PD‐1 blockade based immunotherapy and modulate the malignant progression of multiple tumours by regulating PD‐L1 expression. Upregulation of IRF7 and IRF9 can lead to poorer efficacy of chemotherapy and targeted therapy in breast cancer and melanoma patients, respectively.^20^, ^21^ In addition, research has suggested that IRF family is linked to tumour immune microenvironment (TIME) and prognosis of colorectal cancer.^22^

In this study, we systematically evaluated IRF family in 33 tumour samples, including expression profiles, genomics, clinical features and prognostic value. We found that genetic variation, epigenetic alterations, miRNA and lncRNA networks cause aberrant expression of IRFs and are involved in regulating multiple tumour malignant progression. We then calculated IRF‐score in pan‐cancer and analysed the impact of this score on patient survival and tumour progression. Furthermore, we explored the association between IRF‐score and tumour immune characteristics and revealed the potential value of IRF‐score in predicting therapeutic efficacy of targeted therapy and immunotherapy. It is worthwhile to further investigate IRF family as a predictive marker for tumour therapy.

## 2 METHODS AND MATERIALS

### 2.1 Pan‐cancer data acquisition

A flowchart of this work was displayed in Figure S1. This work included nine transcription factors of IRF family (IRF1‐9). UCSC Xena platform (https://xenabrowser.net/datapages/) was utilized to download RNAseq data, clinical information, somatic mutations (SNVs), copy number segment and DNA methylation data for 33 tumour samples and paraneoplastic tissue samples from The Cancer Genome Atlas (TCGA). IRFs expression in different cancer cell lines was extracted from the Cancer Cell Line Encyclopedia (CCLE) database.^23^ Genomics of Drug Sensitivity in Cancer (GDSC) (https://www.cancerrxgene.org/)^24^ and Cellminer (http://discover.nci.nih.gov/cellminer/)^25^ databases were employed to explore the relevance of drug sensitivity and RNA molecules at the molecular level.

### 2.2 ssGSEA

Single‐Sample Gene‐Set Enrichment Analysis (ssGSEA) calculated enrichment scores for each sample and gene set pair, allowing each sample to receive a ssGSEA score for the corresponding gene set. ssGSEA converted the gene expression profiles of individual samples into gene set enrichment profiles, allowing the characterization of cell states or functions depending on biological processes and pathway activity levels.^26^ In this study, the IRF score, immune cell infiltration score and pathway score were quantitatively elucidated in each tumour sample by means of the GSVA package and three gene sets (Table S1), respectively.^27^

### 2.3 Immune infiltrate estimation

According to the transcriptome expression data, ESTIMATE algorithm was applied to estimate tumour purity based on stromal and immune cell ratios^28^ and the CIBERSORT algorithm to quantify 22 tumour‐infiltrating immune cells. 22 gene expression signature sets of immune cell subtypes (LM22) obtained from Cibersort literature^29^ (Table S2). Immunophenoscore were calculated separately for four representative immunophenotype scores (antigen‐presenting, effector, suppressor and checkpoint) with gene expression z scores in the 0–10 range, where higher scores correlate with higher immunogenicity.^30^ Immunophenoscore was obtained from The Cancer Immunome Atlas (TCIA) (https://tcia.at/home). Tumour immune dysfunction and rejection (TIDE) was performed to assess the potential for tumour immune escape.^31^ Higher TIDE scores indicated worse efficacy of immune checkpoint blockade and shorter post‐treatment survival. TIDE scores were taken from TIDE website (http://TIDE.dfci.harvard.edu/).

### 2.4 Pathway enrichment analysis

‘limma’ was applied to identify differentially expressed genes (DEGs, FDR < 0.05, logFCfilter = 1) between IRF‐score groups.^32^ ‘clusterProfiler’ package^33^ was employed to enrich these DEGs for Gene Ontology (GO)^34^ and Kyoto Encyclopedia of Genes and Genomes (KEGG)^35^ functions. Terms with false discovery rate (FDR) < 0.05 are considered to be meaningful. ‘ggplot2’ was applied for visualizing analysis results.^36^

### 2.5 Survival analysis

Grouped according to IRFs and median IRF‐score, this study compared overall survival (OS) and progression free survival (PFS), disease‐free survival (DFS) and disease‐specific survival (DSS) of patients, respectively. Kaplan–Meier curves and log‐rank tests were performed for investigating whether genes were associated with survival outcomes and time and to compare survival differences.

### 2.6 Statistical analysis

All data were analysed using R software (v4.1.1). The Spearman correlation test was conducted for correlation analysis. Comparisons between two groups were done with Wilcoxon test and comparisons between three or more groups were done with Kruskal–Wallis test. Cox regression analysis was performed to evaluate prognostic impact of variables and clinicopathological characteristics. *p* < 0.05 denotes statistical significance. *p < 0.05, **p < 0.01, ***p < 0.001.

## 3 RESULTS

### 3.1 Expression pattern of IRF family and its prognostic analysis

**FIGURE 1:**
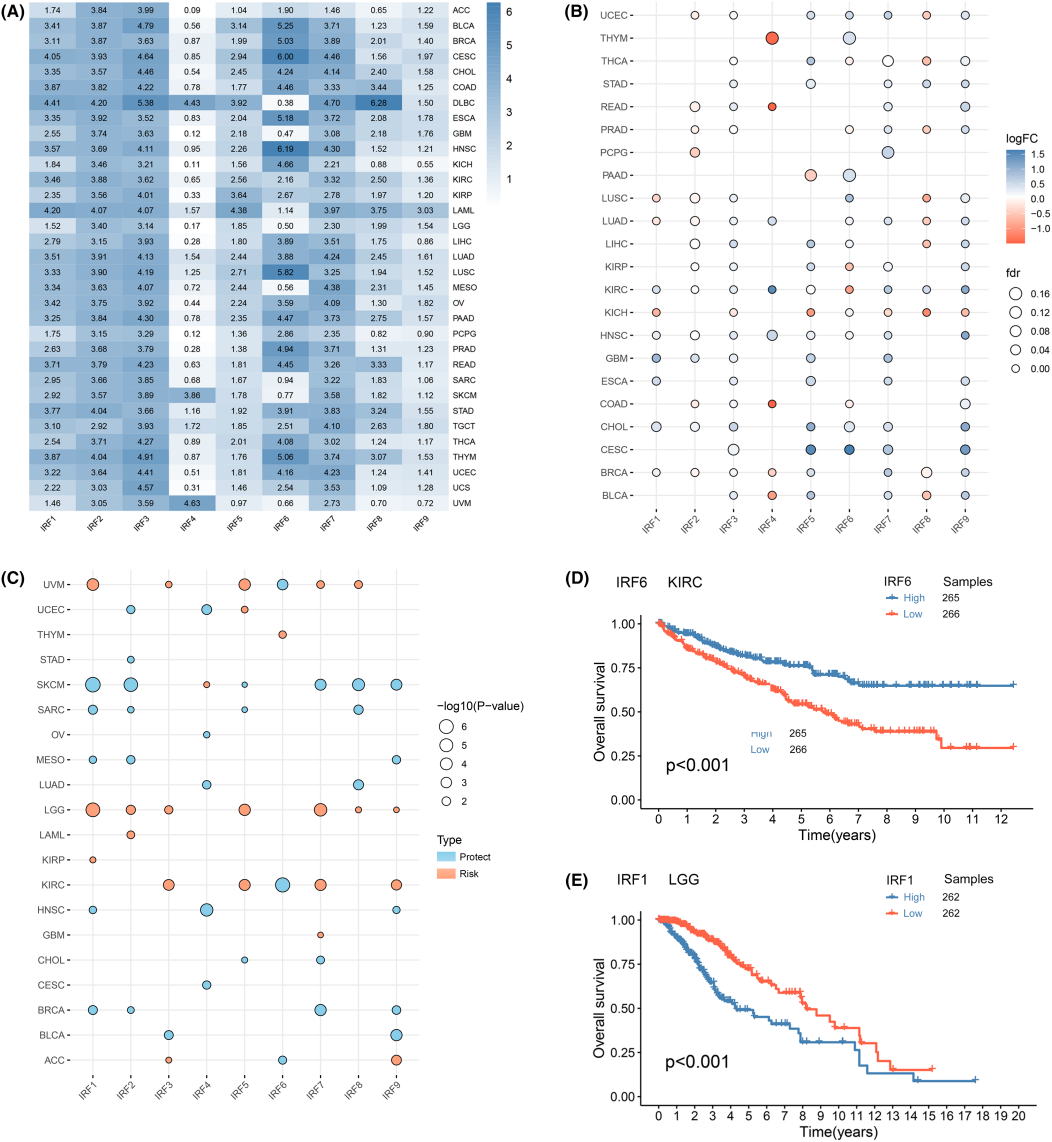
The expression profile and prognostic value of IRF family gene in pan‐cancer. (A) Heat map of IRF family gene expression signatures (Transcripts Per Million, TPM) in TCGA dataset. (B) Differences in mRNA expression between normal and tumour samples. Blue represents high expression in tumours, while red is the opposite. (C) Overall survival analysis of IRFs. Red dots indicate that the IRF can be used as a risk factor, while blue dots indicate a protective factor. The dot size represents the p‐value (from Kaplan–Meier analysis), and the significance of the effect on pan‐cancer survival. (D) Kaplan–Meier curves measure the impact of IRF6 on OS in KIRC patients. (E) Kaplan–Meier curves measure the impact of IRF1 on OS in LGG patients.

To characterize IRF family expression in tumours, we analysed and compared the expression patterns of IRFs in different tissues from TCGA, GTEx and CCLE databases. As seen in Figure 1A; Figures S2A and S2B, IRF1‐9 presented clearly heterogeneous expression in different tissues with a strong tissue specificity. For example, IRF6 was expressed at significantly lower levels in DLBC, GBM, LGG and MESO than multiple tumours such as CESC, HNSC and LUSC. Although IRFs have different expression patterns in tissue samples of different origins, the corresponding expression profiles in tumour tissue, tumour cells and normal tissue were similar. For example, compared to other IRFs, IRF2 and IRF3 were expressed at high levels in most tissues, while the opposite was true for IRF4. We then used TCGA database to explore the differences in IRFs expression in normal and tumour tissues. Multiple IRFs were aberrantly expressed in cancer tissues compared to normal tissues. Most differentially expressed IRFs were upregulated in tumours (*p* < 0.05, Figure 1B; Table S3).

Next, we assessed the prognostic value of IRFs (Figure 1C; Figure S2C–E). For example, IRF1 was a risk factor in KIRP and LGG, where higher expression was linked to poorer OS, whereas in BRCA, HNSC, MESO, SARC and SKCM, IRF1 could act as a protective factor. Survival analysis of two differential genes revealed that high expression of IRF6 was related to a good prognosis for KIRC (*p* < 0 0.001, Figure 1D); whereas high expression of IRF1 was related to a poor prognosis for LGG (*p* < 0.001, Figure 1E). The above results demonstrated that IRFs expression in tumours is tissue‐specific and that its dysregulation may contribute to tumorigenesis.

### 3.2 Landscape of genetic variation and DNA methylation modifications of IRF family

We analysed the SNV and copy number variation (CNV) profiles of IRFs in different cancer subtypes to facilitate investigating the potential mechanisms of tumorigenesis. Overall, IRFs had a low SNV frequency in tumours (Figure S3A). The percentage of mutations in the genes was below 5% in a variety of tumours, except in DLBC where the frequency of mutations in IRF4 and IRF8 was 14% and 8%. The frequency of SNPs in IRFs was slightly higher in UCEC than other tumours.

**FIGURE 2:**
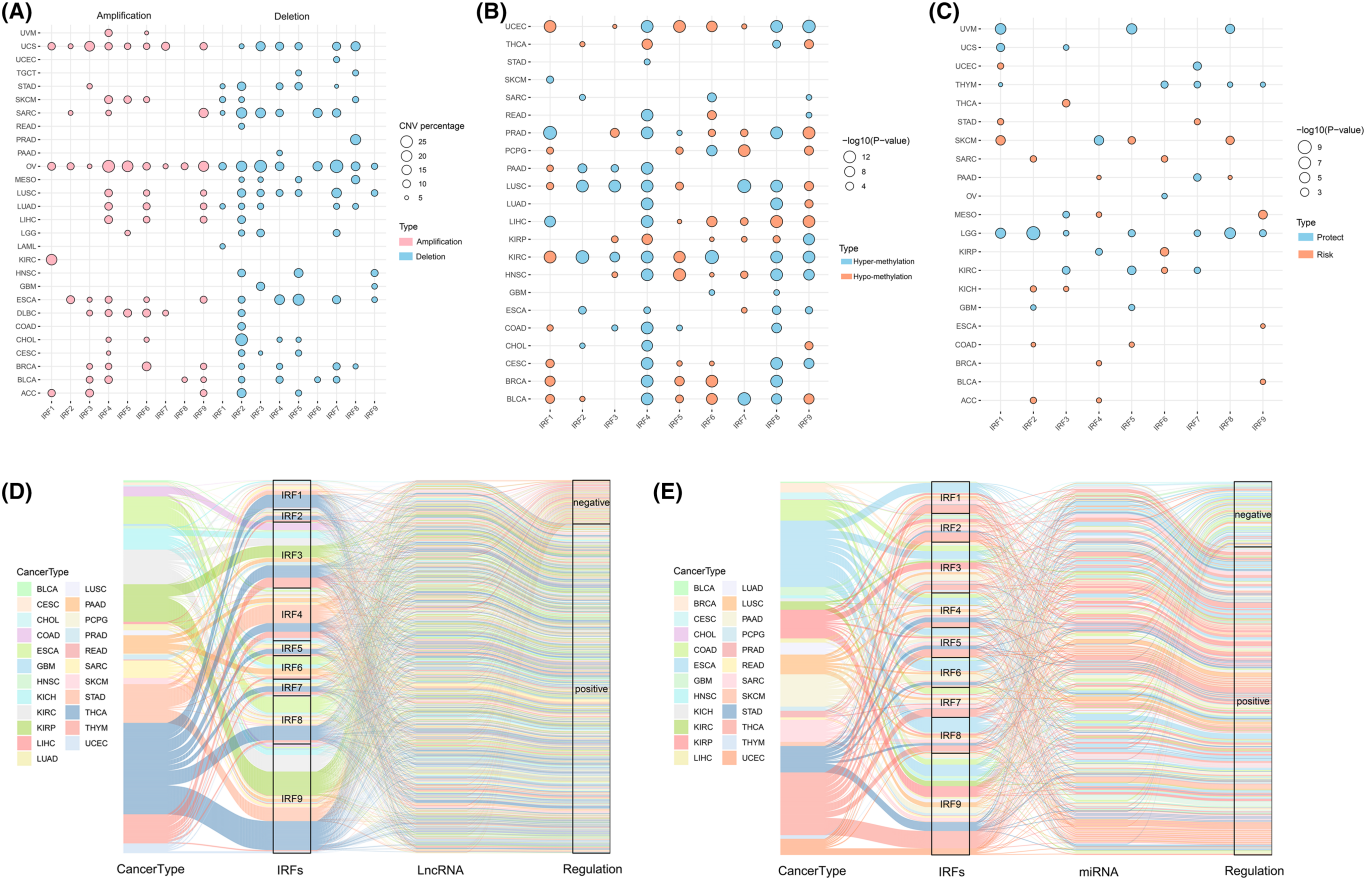
Copy number variation (CNV) and methylation of IRF family gene. (A) Heterozygous CNV profiles display the percentage of heterozygous CNV amplifications and deletions for each gene in the pan‐cancer. Only mutation frequencies greater than 5% are retained. (B) Methylation status of IRF family genes in pan‐cancer. Blue dots represent increased tumour methylation and red dots represent decreased tumour methylation. The size of the dots represents the P‐value for the difference in methylation in tumour and normal samples. (C) Correlation analysis between methylation status of IRF family genes and survival. Patients were differentiated into hypermethylated and hypomethylated based on median values. Red dots represent poor prognosis for hypermethylation and blue dots represent good prognosis for hypermethylation. The size of the dots represents statistical differences. (D, E) Alluvial map demonstrates the regulation of IRF family genes by lncRNAs (D) and miRNAs (E) in pan‐cancer.

Subsequently, we collated and analysed CNV data from IRFs. Analysis of CNV alteration frequencies indicated that CNV alterations were prevalent in multiple tumours in IRFs and copy number deletions were more common (Figure 2A). The occurrence of CNV in IRFs exhibited a clear organ specificity. For example, IRF1 was mostly concentrated in copy number expansions in ACC, KIRC, OV and UCS, whereas deletions were more prevalent in LAML, LUAD, SARC, SKCM and STAD. Figure S3B suggested that CNV changes, as a dichotomous variable, may be associated with aberrant expression of IRFs and tumorigenesis.

Considering the essential role of epigenetic modifications in regulating gene expression, we analysed the DNA methylation profiles of IRFs in pan‐cancer. IRFs had different DNA methylation patterns in different tumours, with a high degree of heterogeneity (Figure 2B). For example, IRF4 exhibited promoter hypomethylation in KIRP and THCA, no significant changes in promoter methylation in GBM, PCPG, SARC and SKCM, and high methylation levels in several tumours including BLCA, BRCA, CESC and COAD. Correlation analysis indicated that promoter methylation of the majority of IRFs could repress gene expression, with a negative correlation between the two (Figure S3C). However, in some tumours, promoter methylation did not fully determine gene expression, although there was a clear correlation between the two. For example, IRF3 promoter hypermethylation should be accompanied by reduced gene expression, whereas in KIRC, IRF3 expression is elevated. This meant that gene expression is not regulated singularly, but is a process in which multiple factors act together. Methylation survival analysis revealed that hypermethylation of most IRFs was related to a good prognosis in LGG, THYM, UVM and KIRC. In tumours such as SKCM, KICH and ACC, the opposite was true (Figure 2C).

### 3.3 IRFs‐related lncRNAs and miRNAs

To elucidate the regulation of IRFs by lncRNAs and miRNAs, we created separate lncRNA and miRNA‐related alluvial maps to visualize the possible regulation of IRFs. As seen in Figure 2D,E, IRFs regulated by lncRNAs accounted for the highest proportion of THCA, and those regulated by miRNAs were most abundant in THCA and ESCA. Compared to other IRFs, lncRNAs and miRNAs were most involved in regulating IRF9 expression, followed by IRF3 and IRF4, and most of these lncRNAs or miRNAs could positively regulate IRFs expression (lncRNA: *R* > 0.4, *p* < 0.001; miRNA: *R* > 0.2, *p* < 0.01). These results indicated that lncRNAs and miRNAs may be involved in regulating tumour malignant progression by modulating IRFs expression.

### 3.4 Expression profile and prognostic performance of IRF‐score in pan‐cancer

**FIGURE 3:**
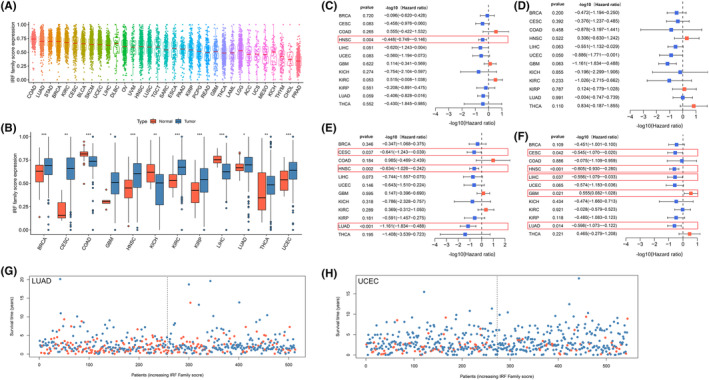
Expression and prognostic landscape of IRF‐score in pan‐cancer. (A) Expression levels of IRF scores in 33 cancers. (B) Expression levels of IRF scores in tumour and normal tissues. (C–F) Survival analysis of IRF scores in pan‐cancer, including OS (C), DFS (D), DSS (E) and PFS (F). HR values were log10‐transformed. (G, H) Scatter plot of the distribution of OS with IRF score in LUAD (G) and UCEC (H) patients. Red dots indicate dead samples, blue dots indicate surviving samples. *p < 0.05, **p < 0.01, ***p < 0.001.

Based on the ssGSEA algorithm, we calculated IRF‐related scores (IRF‐score) in 33 tumour samples. All cancers showed a broad IRF‐score. Among them, IRF‐score had highest average expression in COAD and the lowest in PRAD (Figure 3A). Subsequently, we compared the differences in the expression of IRF‐score in normal and tumour samples. The results displayed that there were significant differences in the expression of IRF‐score in 12 normal and tumour samples (Figure 3B). IRF‐score expression was significantly higher in BRCA, CESC, GBM, HNSC, KIRC, KIRP, LUAD, THCA and UCEC than in normal samples, while the reverse was evident in COAD, KICH and LIHC (*p* < 0.05).

Based on the median expression of IRF‐score in the above 12 tumours, we classified patients into high and low score groups to facilitate the exploration of the clinical value of IRF‐score. Univariate COX regression analysis indicated that IRF‐score had different prognostic performance in different tumours. IRF‐score was related to OS in HNSC, DFS in UCEC, DSS in CESC, HNSC, LUAD and PFS in CESC, HNSC, LIHC, LUAD and could be a protective factor for survival in these tumours (Figure 3C–F, *p* < 0.05, HR <1). In contrast, in GBM, IRF‐score could be regarded as a risk factor for its PFS (Figure 3F, *p* < 0.05, HR >1). Kaplan–Meier curves further demonstrated survival differences between high and low groups in different tumours (Figure S4A, *p* < 0.05). For most tumours, the higher the IRF‐score, the more positive the prognosis of the patient. The potential value of IRF‐score in predicting survival in LUAD and UCEC was higher compared to other tumours. Therefore, we further explored the correlation between IRF‐score and clinicopathological characteristics of the two tumours. Evidently, the lower the IRF‐score, the lower the probability of survival of the patient (Figure 3G,H; Figure S4B) and the later the staging classification of the patient (Figure S4C). These results suggested that IRF‐score has broad application in predicting prognosis in multiple tumours, particularly in LUAD and UCEC.

### 3.5 Analysis of IRF‐score‐related pathways and their functions

**FIGURE 4:**
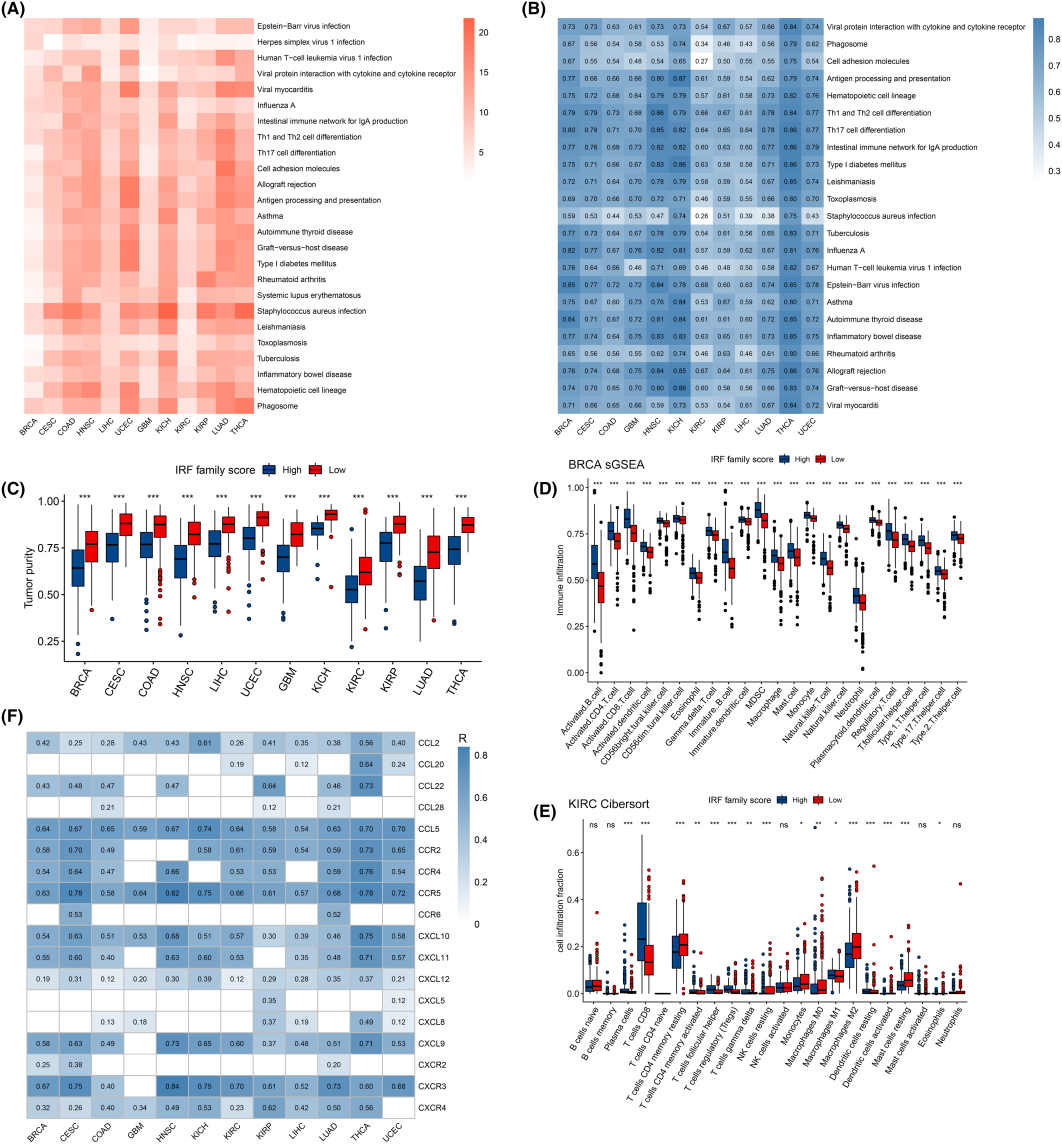
Pathway and immune infiltration analysis of IRF scores in pan‐cancer. (A) Correlation between IRF scores and KEGG cross pathways. The colour represents the *p* value, the darker the colour, the more statistically significant the correlation between the score and the pathway. (B) Correlation between IRF score and KEGG pathway score (ssGSEA score). The colour represents the correlation, the darker the colour, the stronger the correlation between the score and the pathway. (C) Differences in tumour purity in high and low IRF score groups. (D) Abundance of each immune cell in both IRF scoring groups in BRCA (ssGSEA). (E) Ratio of infiltration of each immune cell (cibersort) in both IRF scoring groups in KIRC. The proportion of all cells infiltrated was added to 1. (F) Heat map of the correlation between IRFscore and multiple chemokines. *p < 0.05, **p < 0.01, ***p < 0.001.

To investigate the potential biological functions of IRF‐score in different tumours, we screened 12 tumours for differentially expressed genes between high and low groups and performed GO and KEGG enrichment analyses. In the GO enrichment analysis, we described the function of IRF‐score in BP (Biological process), MF (Molecular Function) and CC (Cellular Component), respectively. We found that IRF‐score was closely correlated with multiple immune‐related pathways in pan‐cancer, such as immune response‐activating signal transduction, B cell mediated immunity (Table S4). In contrast, KEGG enrichment results revealed that IRF‐score is mostly linked to several immune diseases, infectious diseases and the immune system, such as Asthma, Viral myocarditis and Th17 cell differentiation (Figure 4A). We then looked up these pathway‐related genes from the KEGG website and calculated each pathway score to quantify the activity of these pathways and the correlation with IRF‐score. We first compared the pathway scores between two groups and observed that the activity of these pathways was inhibited in low score group (Figure S5A). Correlation analysis similarly indicated that these pathway scores were positively correlated with IRF‐score, with a subsequent increase in pathway activity as IRF‐score increased (Figure 4B).

### 3.6 Analysis of immune infiltration in different IRF‐score groups

Enrichment analysis revealed that IRF‐score is involved in multiple immune pathways, so we further explored the impact of IRF‐score on pan‐cancer TIME. First, we applied estimate algorithm to calculate the proportion of stromal and immune cells in each tumour sample and to assess tumour purity. Evidently, among the 12 tumours, immune cells and stromal cells were most abundant in high IRF‐score group, which represents low tumour purity in high‐scoring group (Figure 4C; Figure S5B). Subsequently, we applied ssGSEA and Cibersort algorithms to assess the level of infiltration of different immune cells and the percentage, respectively. As before, we calculated the ssGSEA enrichment scores of immune cells in each tumour sample and visualized the analysis. It was apparent that almost all immune cells were more significantly enriched in high IRF‐score group (Figure 4D; Figure S6A). Composition analysis indicated that CD8 T cells, resting memory CD4 T cells, M0 macrophages and M2 macrophages were generally overrepresented in 12 tumours (Figure 4E; Figure S6B–L). Interestingly, we found that in tumours where IRF‐score could be a protective factor such as LIHC, LUAD, HNSC, UCEC, CESC and THCA, effector immune cells represented by CD8^+^ T cells were significantly more infiltrated in high score group, while immunosuppressive cells represented by M2 macrophages were mostly enriched in low IRF‐score (Figure S6B–G). Differently, the proportion of these immune cells infiltrating the GBM for which IRF‐score was a risk factor was reversed (Figure S6H). This result confirmed, from the side, that IRF‐score is reliable in predicting tumour prognosis.

Chemokines have been studied to direct the migration of immune cells to initiate and deliver an effective anti‐tumour immune response.^37^ (Table S5) We next explored the correlation between IRF‐score and chemokines and their receptors. It was evident that IRF‐score is strongly associated with multiple chemokines and receptors in 12 tumours, in particular CCL5, CCR2/4/5, CXCL9/10/11 and CXCR3 (*p* < 0.05, Figure 4F). Strikingly, the immune cells targeted by these chemokines did infiltrate more in high‐score groups. These results indicated that differences in the types and levels of various chemokines may be one of the main reasons for the differences in tumour‐infiltrating immune cells in different score groups. Based on immune cell infiltration characteristics, and in conjunction with previous studies,^38^, ^39^ we concluded that among the 12 tumours high IRF‐score group corresponds mainly to hot tumours, characterized by heavily infiltrated activating immune cells. In contrast, low IRF‐score group corresponded to cold tumours, with fewer infiltrating immune cells and more immunosuppressive cells.

### 3.7 Targeted drug sensitivity analysis

**FIGURE 5:**
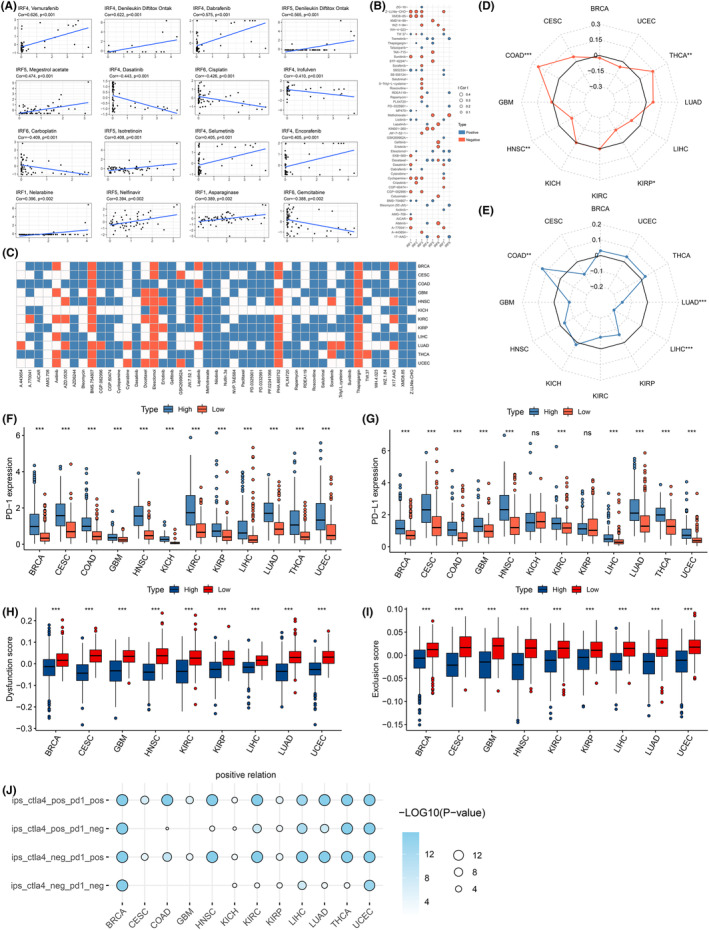
Analysis of IRF scores in pan‐cancer drug therapy. (A) Scatter plot shows the correlation between IRF family gene expression and different drugs (Pearson correlation coefficient, Cellminer). (B) Correlation between IRF1‐8 and tumour drug sensitivity (GDSC). The size of the points depends on the absolute value of the correlation coefficient. Blue represents a positive correlation between IRF expression and sensitivity, while red represents a negative correlation. *p*‐values for all points are less than 0.05. (C) Heat map of sensitivity of different oncology drugs in pan‐cancer in high and low scores (GDSC). Blue represents a lower IC50 in the high scoring group; while red represents a lower IC50 and better drug efficacy in the low scoring group. (D, E) Radar plot of the correlation between IRF scores and MSI (**D**) or TMB (E). (F, G) Differences in PD‐1 (F) and PD‐L1 (G) expression in high and low IRF groups. (H, I) Differences in TIDE scores, including dysfunction score (H) and exclusion score (I), among high and low IRF groups. (J) Differences between the 4 IPS scores and in the two scoring groups. Blue represents high IPS in the high IRFscore group, and the colour and size of the ball represent the *p* value for the difference in IPS in two groups. *p < 0.05, **p < 0.01, and ***p < 0.001.

To investigate the impact of IRFs on oncology drug sensitivity, we explored drug sensitivity and RNA molecular correlations by performing a comprehensive analysis of two pharmacogenomic databases, CellMiner and GDSC. First, we calculated the correlation between IRFs expression and different drug sensitivities and listed the top 16 drugs with the strongest correlation (Cellminer, *p* < 0.05, Figure 5A and Table S6). The results revealed that sensitivity (IC50) to most drugs, such as Estramustine, Fluphenazine, Hydroxyurea and Isotretinoin, was mostly positively correlated with IRF2/4/5/8 expression. In contrast, IRF1/6 expression was negatively related to sensitivity to various drugs, including Arsenic trioxide, Asparaginase, Bleomycin, BMN‐673 and Carboplatin. Furthermore, we obtained and analysed IRFs drug sensitivity information from the GDSC database. Again, we observed a significant correlation between the sensitivity of several drugs and IRFs expression (Figure 5B, *p* < 0.05). Typically, Cyclopamine and SB52334 drug sensitivities appeared negatively and positively correlated with the expression of IRF1/2/3/7, respectively. Subsequently, based on IRFs‐sensitive compounds screened by GDSC, we predicted the therapeutic response to these drugs in different IRF‐score groups. For the vast majority of drugs, high‐scoring groups were more sensitive to these drugs (Figure 5C; Figure S7A). These results implied that dysregulated IRFs expression might play a critical role in the body's response to targeted drugs.

### 3.8 Predicting immunotherapy response in IRF‐score group

Immunotherapy refers to the removal of cancer cells by increasing the recognition of tumours by immune cells in the body. Among them, tumour immune checkpoint inhibitors (ICIs) have the most mature clinical research and are the most widely used.^40^ However, in most cancers, only about one‐third of patients respond to ICIs. Researchers have identified multiple factors that may influence the efficacy of ICIs treatment, including the degree of cytotoxic T cell infiltration, mutation or neoantigen load, PD‐1/L1 levels, and mismatch repair defects.^41^, ^42^, ^43^, ^44^ MSI, TMB and PD‐1/PD‐L1 expression are currently considered as markers for screening patients for immunotherapy, and their expression all positively correlated with immunotherapy efficacy. In 12 tumours, we observed that IRF‐score related to MSI in four cancer types and TMB in three cancer types. IRF scores positively correlated with MSI and TMB values for multiple tumours (*p* < 0.05, Figure 5D,E). We then used ‘survminer’ package to calculate the optimal cut‐off value of TMB in each cancer and analyse TMB's prognostic performance. We observed that in KIRP and UCSC, patients with high TMB exhibited better survival. Conversely, in BRCA, HNSC, KICH, KIRC, LIHC and THCA, patients with low TMB had a more optimistic prognosis (Figure S7B, *p* < 0.05). Considering the contraindicated prognostic value of TMB and IRF‐score, we assessed the synergistic effect of two scores in prognostic stratification. Stratified survival results displayed that TMB‐ and IRF‐score based tests did not interfere with each other. IRF‐score subtypes exhibited survival differences in high and low TMB subgroups consistent with their independent predictive results (Figure S7C). Next, we analysed the correlation between multiple immune checkpoints, including PD‐1/L1, and IRF‐score. We observed a highly positive relationship between IRF‐score and immune checkpoints in most tumours (*p* < 0.05, Figure S8A). PD‐1 and PD‐L1 expression levels were significantly higher in high scoring group (Figure 5F,G). These results pointed to the possibility that higher IRF‐score patients may benefit more from immunotherapy.

Apart from the above indicators, we additionally applied TIDE score and tumour stemness score to predict ICIs treatment effects. First, we assessed dysfunction score and exclusion score expression in different tumours in high and low IRF‐score groups, respectively. Evidently, both scores of TIDE were consistently lower in high IRF‐score group among 12 tumours (Figure 5H,I). A higher TIDE score represents a higher likelihood of immunosurveillance escape within the tumour and a poorer outcome of immunotherapy. Therefore, from the perspective of TIDE scores, high IRF‐score group was more likely to receive immunotherapy. Subsequently, we evaluated tumour cell stemness scores (DNAss and RNAss) distribution in different scoring groups. The results displayed that DNAss and RNAss scored higher in low IRF‐score group (*p* < 0.05, Figure S8B,C), which indicated that tumour stem cells in low‐scoring group were more active, less differentiated and less responsive to treatment. Given these results, we concluded that high IRF‐score patients are sensitive to immunotherapy. To test this hypothesis, we calculated immunophenoscore in 12 tumours and predicted the patient's response to ICIs. We noticed that all four different immunophenoscore were higher in high IRF‐score group, which meant that high IRF‐score group was more effective for immunotherapy (Figure 5J).

## 4 DISCUSSION

IRFs are proven to act as prognostic markers for tumours and perform essential roles in regulating malignant progression and immune infiltration. However, there has been no systematic pan‐cancer analysis of the prognostic performance, immune infiltration and pharmacological applications of entire IRF family. Therefore, the present study provides a systematic analysis of IRF family in 33 tumour samples, including expression differences, genetic alterations, immune infiltration and drug sensitivity.

In this study, we utilized TCGA database to discuss IRF1‐9 expression in pan‐cancer and its prognostic value. We identified IRFs that were significantly differentially expressed in multiple tumour and normal samples with tumour specificity. These differentially expressed IRFs could be actively involved in regulating tumour development as protective or risk factors for tumour prognosis. To further investigate the potential oncogenic mechanisms of IRFs, we comprehensively assessed the genomic features of IRFs in pan‐cancer, including SNV, CNV and methylation modifications. Genetic analysis revealed a low frequency of mutations but a high incidence of copy number variants in IRFs in pan‐cancer. Combined with expression analysis, a positive correlation was detected between CNV and expression of most IRFs in pan‐cancer, and their copy number alterations could promote tumorigenesis by regulating gene expression levels. For example, high SNP of IRF4 in DLBC and the copy number homogeneity deficiency of IRF2 in LIHC could both be risk factors for DLBC and LIHC development, which is consistent with previous studies.^45^, ^46^ Previous epigenetic studies have demonstrated that DNA methylation attenuates gene expression levels.^47^ In this study, we found that hypermethylation of most IRFs downregulates gene expression, thereby promoting or suppressing tumour malignant progression. However, DNA hypermethylation does not necessarily silence gene expression. For example, in KIRC, IRF3 hypermethylation was accompanied by elevated gene expression, which was contrary to previous studies. For one thing, recent studies revealed that promoter DNA hypermethylation induces gene expression^48^; for another, gene expression was affected by several factors. In lncRNA/miRNA‐mRNA interaction network, we observed that IRF3 and IRF9 were predominantly regulated by lncRNA and miRNA targets, and lncRNA/miRNA mostly positively regulated IRFs expression. We therefore hypothesized that IRFs inheritance, altered expression genetics and lncRNA/miRNA interaction networks could collectively regulate IRFs expression and participate in tumorigenesis.

To further characterize IRFs biology in pan‐cancer, we constructed IRF‐score based on ssGSEA and identified that IRF‐score were significantly more expressed in BRCA, CESC, GBM, HNSC, KIRC, KIRP, LUAD, THCA and UCEC than normal samples, while the opposite was true in COAD, KICH and LIHC. We then assessed associations between IRF‐score and survival in 12 tumours. COX and K‐M analyses revealed that IRF‐score were remarkably linked to survival and clinicopathological features in multiple tumours, particularly UCEC and LUAD. Pathway function analysis revealed that IRF‐score were associated with multiple immune pathways. Given IRFs' important role in regulating host immune responses and TIME,^49^, ^50^ we further discussed the correlation between IRF‐score and immune cells in pan‐cancer TIME.

It has been shown that immune cells are the cellular basis of immunotherapy. Some studies have found that the correlation between IRFs and immune cells has an impact on immunotherapy outcomes For example, basic leucine zipper ATF‐like transcription factor (BATF) and IRF4 have been found to synergistically counteract T cell exhaustion and increase effector function to improve antitumor responses.^51^ Tumour infiltrating lymphocyte counts, PD‐L1 positivity and expression of the interferon regulatory genes IRF1, 4, 7 are synergistically downregulated in breast cancer metastases with a lower predictive profile for immunotherapy response.^52^ Obviously, in this research, most immune cells were more significantly enriched in high IRF‐score group. In particular, in tumours where IRF‐score could be a protective factor, effector immune cells, represented by CD8^+^ T cells, were significantly more infiltrated in high‐score group, while immunosuppressive cells, represented by M2 macrophages, were mostly enriched in low IRF‐score. The opposite ratio of infiltration of these cells in tumours with IRF‐score as a risk factor was observed. Moreover, we discovered that certain chemokines mostly infiltrated in high scoring group. Chemokines guide immune cell migration in TIME, resulting in an immune signature of microenvironment.^37^ Therefore, we propose that the different types and levels of various chemokines may be one of the main reasons for the differences in tumour‐infiltrating immune cells in different scoring groups. Based on the immune cell infiltration characteristics, combined with previous studies,^38^, ^39^ we concluded that among these 12 tumours high IRF‐score group corresponds to hot tumours with higher infiltration of effector immune cells, while low IRF‐score group corresponds to cold tumours described as having fewer effector immune cells and more immunosuppressive cells.

From 1997, when rituximab was the first monoclonal antibody‐targeted drug approved for the treatment of tumours, a new era of oncology treatment was introduced—targeted therapy.^53^ Unlike other drugs, targeted drugs are endowed with the ability to target specific lesions and accumulate or release active ingredients at the target site, creating relatively high concentrations at the target site, increasing their efficacy while suppressing toxic side effects and reducing damage to normal tissues and cells.^54^ In this study, based on two major pharmacogenomic databases, Cellminer and GDSC, we discovered that multiple IRFs and IRF‐score can be involved in regulating drug sensitivity in pan‐cancer. For most drugs, higher scoring groups are more sensitive to these drugs and may have better treatment outcomes. Besides, Guo et al. found that IRF2 could increase resistance to lenvatinib in hepatocellular carcinoma patients.^55^ IRF4 inhibition enhanced the sensitivity of myeloma cells to standard cancer therapeutic agents.^56^ Therefore, we hypothesized that IRFs could not only predict tumour patients' therapeutic effect on targeted drugs, but also target IRFs for cancer treatment.

ICIs are among the hottest recent oncology therapeutics, including inhibitors targeting PD‐1, PD‐L1 and CTLA‐4.^57^ These drugs loosen up immune cells that are suppressed by tumour cells, allowing effector immune cells to attack tumour cells.^58^ Patients can now be evaluated for immunotherapy‐related indicators, including PD‐L1, TMB and MSI, before immunotherapy is administered, both to exclude patients with tumours that may benefit less and to analyse some biomarkers that can predict the efficacy of immunotherapy drugs.^39^, ^41^, ^42^, ^43^ Analysis of multiple biomarkers has consistently found that immunotherapy works better in high IRF‐score patients. We concluded that IRF‐score are potential biomarkers for the effectiveness of immunotherapy in oncology patients. Therefore, our findings could provide a basis for subsequent in‐depth work related to cancer drug therapy research.

However, this study still has many shortcomings. The conclusions obtained in this manuscript were based on one database, TCGA, and there is a lack of other databases to validate the conclusions obtained. Considering the high variability among different tumours, for this reason, more clinical data are needed to support the conclusion on the prognostic performance of IRF scores on different tumours, which will be further analysed and discussed in subsequent studies. In addition, in clinical practice, patient treatment regimens and outcomes are affected by tumour heterogeneity and individual differences, and this study still lacks an independent database of clinical samples to further analyse the conclusions.

## 5 CONCLUSION

Overall, we comprehensively analysed the genomic and clinical profiles of IRF family in pan‐cancer and constructed IRF‐score to further analyse IRFs in immune infiltration and drug therapy. We discovered that IRF‐score could be used to predict the efficacy of several oncology drug treatments, including targeted therapies and immunotherapy. Taken together, this is the first study to comprehensively analyse IRF family in pan‐cancer, particularly in drug therapy, and could point the way to achieving personalized oncology treatment.

## AUTHOR CONTRIBUTIONS

**Hua‐Guo Xu:** Funding acquisition (lead); investigation (lead). **Can Chen:** Methodology (lead); writing – original draft (lead); writing – review and editing (equal). **Lin‐Yuan Chen:** Data curation (lead); methodology (equal); writing – original draft (equal); writing – review and editing (supporting). **shiyang Pan:** Project administration (lead); validation (lead); visualization (lead).

## FUNDING INFORMATION

This study was supported by Natural Science Foundation of Jiangsu Province of China (BK20181492), the National Key Clinical Department of Laboratory Medicine of China in Nanjing, Key laboratory for Laboratory Medicine of Jiangsu Province ZDXKB2016005, ZDXK202239 and by the Priority Academic Program Development of Jiangsu Higher Education Institutions.

## CONFLICT OF INTEREST STATEMENT

None.

## Supporting information


Figure S1.
Figure S2.
Figure S3.
Figure S4.
Figure S5.
Figure S6.
Figure S7.
Figure S8.

Table S1.

Table S2.

Table S3.

Table S4.

Table S5.

Table S6.

## Data Availability

The data that support the findings of this study are available from the corresponding author upon reasonable request.
