# Supplementary material for: Pan‐cancer analysis identifies the IRF family as a biomarker for survival prognosis and immunotherapy
Source: J Cell Mol Med. 2023 Dec 21;28(3):e18084. doi: 10.1111/jcmm.18084 (PMC10844690; doi:10.1111/jcmm.18084)
Supplement: Supplementary file 1 — Figure S1. Figure S2. Figure S3. Figure S4. Figure S5. Figure S6. Figure S7. Figure S8. [file JCMM-28-e18084-s002.pdf]

Supplementary Appendix

This appendix has been provided by the authors to give readers additional information about their work. Supplement to: Pan-cancer analysis identifies the IRF family as a biomarker for survival prognosis and immunotherapy

Pan-cancer analysis identifies the IRF family as a biomarker for survival prognosis and immunotherapy

Supplemental Materials

Table of Contents

Figure S1 Flowchart of this work.
Figure S2 Expression and survival profiles of IRFs in pan-cancer.
Figure S3 Single nucleotide variation (SNV) frequency, CNV and methylation of IRFs.
Figure S4 Survival and clinical analysis of IRF-score in pan-cancer.
Figure S5 Pathway and immuno-infiltration analysis of IRF scores in pan-cancer.
Figure S6 ssGSEA and Cibersort analysis of IRF scores in pan-cancer.
Figure S7 Correlation between IRFscore and drug sensitivity and TMB.
Figure S8 Correlation between IRF-score and immune checkpoints and tumour stem cells.

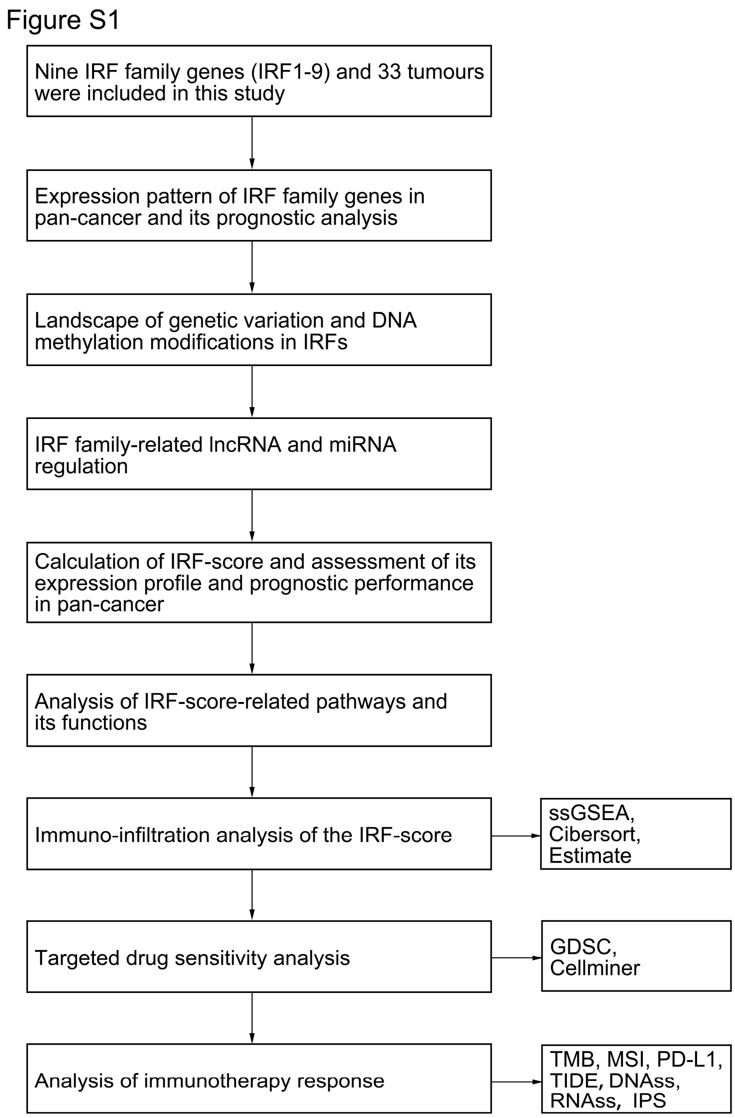

Figure S1 Flowchart of this work.

Figure S2

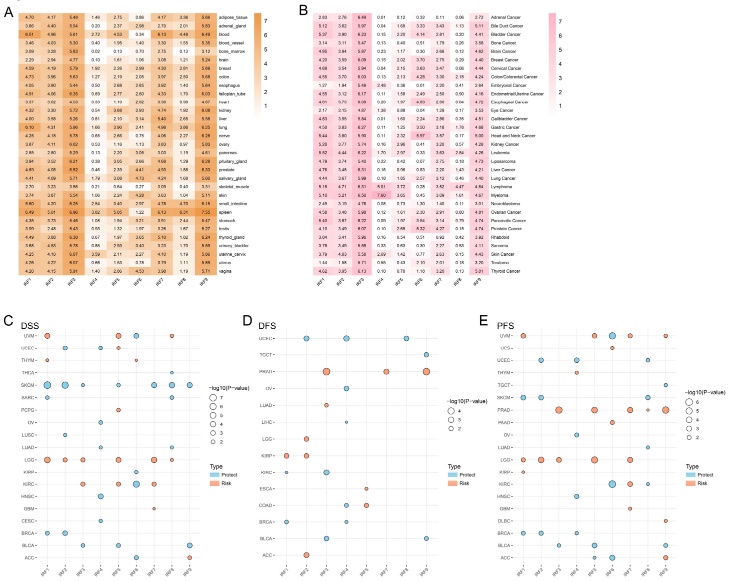

Figure S2 Expression and survival profiles of IRFs in pan-cancer. **A-B**. Heat map of IRF family gene expression signatures in GTEx (**A**) and CCLE (**B**) dataset. **C-E**. Survival analysis of IRF, including disease-specific survival (**C**), disease free survival (**D**) and progression free survival (**E**).

Figure S3

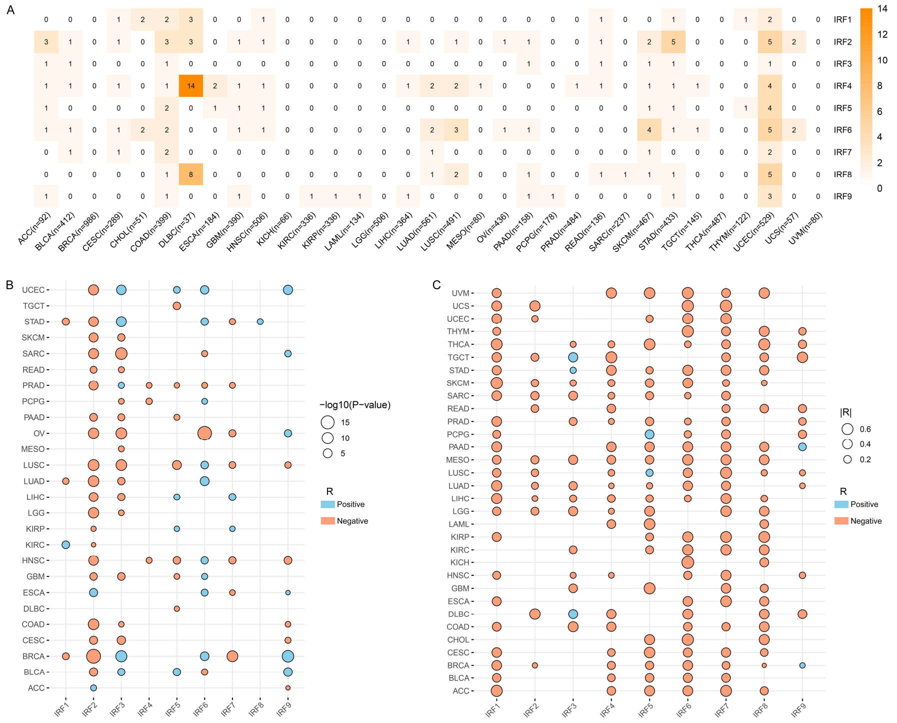

Figure S3 Single nucleotide variation (SNV) frequency, CNV and methylation of IRFs. **A.** Frequency of mutations in the IRF family. The numbers represent the number of samples with the corresponding mutated IRF in respective tumors. **B.** Correlation of CNV with mRNA expression. (Person correlation coefficient). The size of the dots represents statistical significance. **C.** Correlation of methylation with mRNA expression. The size and colour of the dots represent correlation. Orange represents negative correlation; blue represents positive correlation.

Figure S4

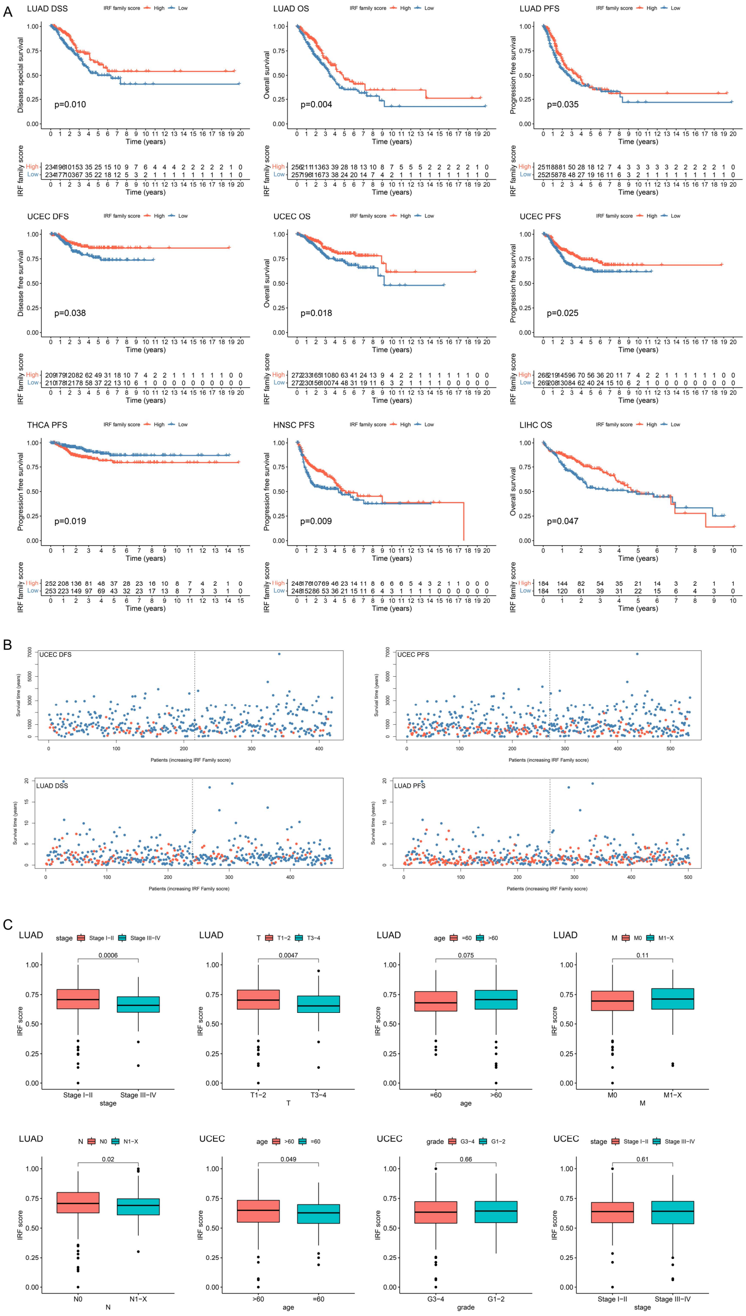

Figure S4 Survival and clinical analysis of IRF-score in pan-cancer. **A.** Kaplan-Meier analysis of survival differences in IRF-score in pan-cancer. **B.** Scatter plot of the distribution of DSS, DFS and PFS with IRF score in LUAD and UCEC patients. **C.** Boxplots of IRF-score between different characteristics in pan-cancer patients, including patient age, stage, grade, T and M.

Figure S5

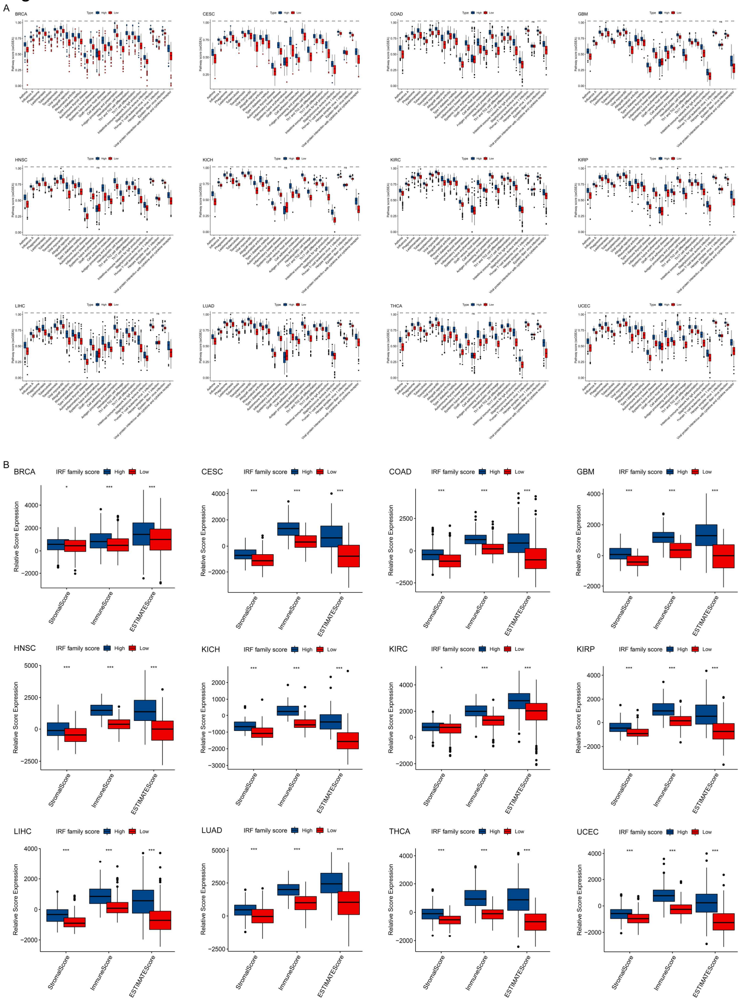

Figure S5 Pathway and immuno-infiltration analysis of IRF scores in pan-cancer. **A.** Infiltration abundance of each pathway score (ssGSEA) in two IRF scoring groups in pan-cancer. **B.** Results of ESTIMATE calculations in two IRF score groups in pan-cancer, including stromal score, immune score and estimate score.

Figure S6

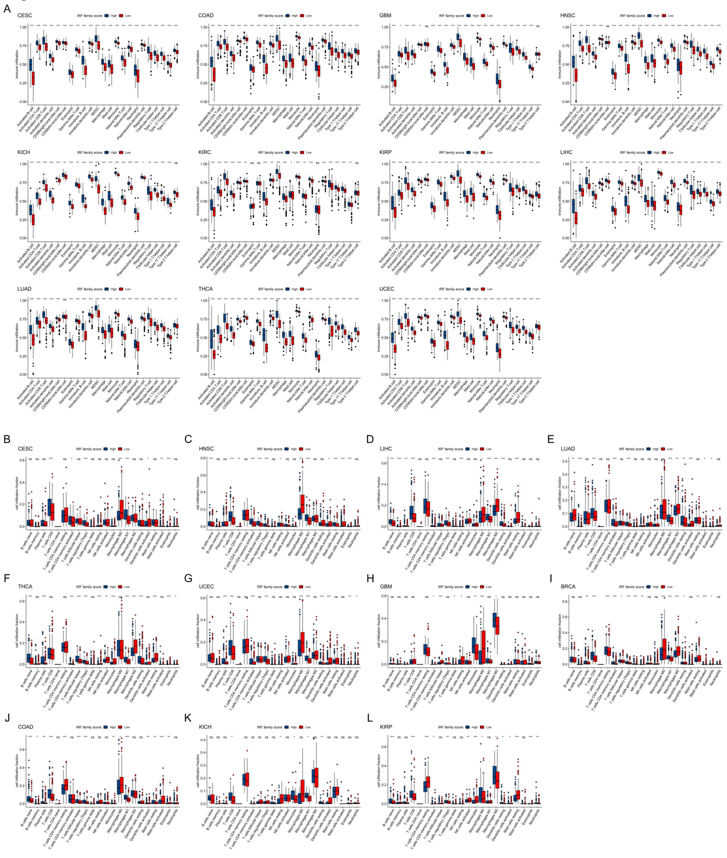

Figure S6 ssGSEA and Cibersort analysis of IRF scores in pan-cancer. **A**. Infiltration abundance of each immune cell in two IRF scoring groups in pan-cancer(ssGSEA). **B-L.** Ratio of infiltration of each immune cell in both IRF scoring groups in pan-cancer (Cibersort).

Figure S7

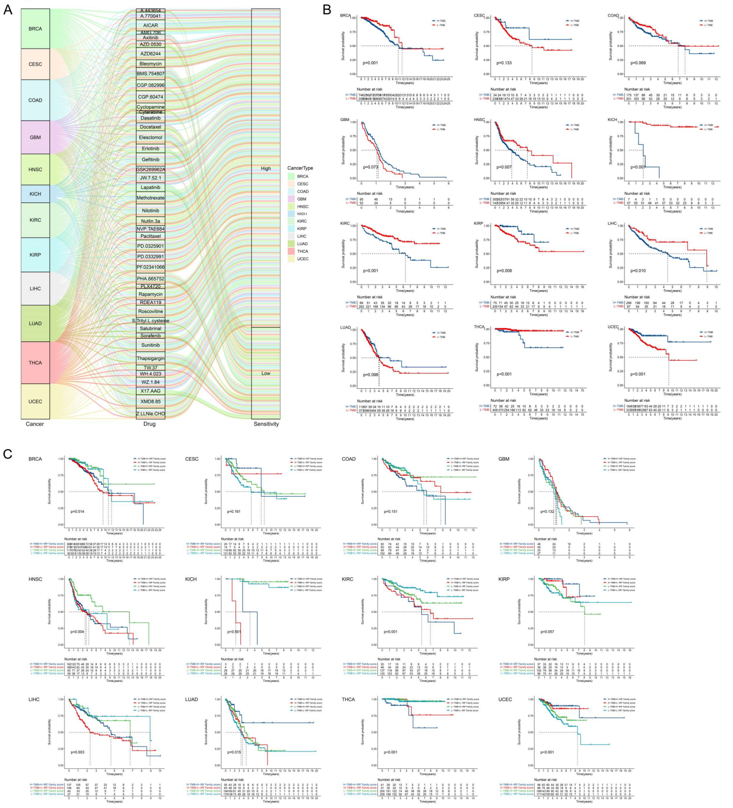

Figure S7 Correlation between IRFscore and drug sensitivity and TMB. **A.** Alluvial plots show the sensitivity distribution of different drugs in pan-cancer in two subgroups. **B.** Kaplan-Meier curves for high and low TMB groups in pan-cancer. **C.** Kaplan-Meier curves for patients stratified by TMB and ICI scores in pan-cancer.

Figure S8

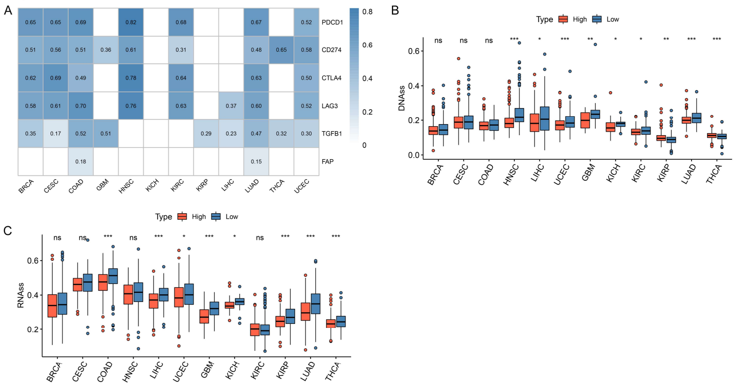

Figure S8 Correlation between IRF-score and immune checkpoints and tumor stem cells. **A.** Heat map of the correlation between IRF-score and immune checkpoints. **B.** Differences in DNAss distribution among high and low IRF score groups in pan-cancer. **C.** Differences in RNAss distribution among high and low IRF score groups in pan-cancer.
